# Overexpression of the autism candidate gene *Cyfip1* pathologically enhances olivo-cerebellar signaling in mice

**DOI:** 10.3389/fncel.2023.1219270

**Published:** 2023-07-20

**Authors:** Silas E. Busch, Dana H. Simmons, Eric Gama, Xiaofei Du, Francesco Longo, Christopher M. Gomez, Eric Klann, Christian Hansel

**Affiliations:** ^1^Department of Neurobiology, The University of Chicago, Chicago, IL, United States; ^2^Department of Neurology, The University of Chicago, Chicago, IL, United States; ^3^Center for Neural Science, New York University, New York, NY, United States; ^4^Institute for Neuroscience and Physiology, University of Gothenburg, Gothenburg, Sweden

**Keywords:** autism, cerebellum, Purkinje cell, CYFIP1 OE transgenic, electrophysiology, two-photon Ca^2+^ imaging

## Abstract

*Cyfip1*, the gene encoding cytoplasmic FMR1 interacting protein 1, has been of interest as an autism candidate gene for years. A potential role in autism spectrum disorder (ASD) is suggested by its location on human chromosome 15q11-13, an instable region that gives rise to a variety of copy number variations associated with syndromic autism. In addition, the CYFIP1 protein acts as a binding partner to Fragile X Messenger Ribonucleoprotein (FMRP) in the regulation of translation initiation. Mutation of *FMR1*, the gene encoding FMRP, causes Fragile X syndrome, another form of syndromic autism. Here, in mice overexpressing CYFIP1, we study response properties of cerebellar Purkinje cells to activity of the climbing fiber input that originates from the inferior olive and provides an instructive signal in sensorimotor input analysis and plasticity. We find that CYFIP1 overexpression results in enhanced localization of the synaptic organizer neurexin 1 (NRXN1) at climbing fiber synaptic input sites on Purkinje cell primary dendrites and concomitant enhanced climbing fiber synaptic transmission (CF-EPSCs) measured using whole-cell patch-clamp recordings from Purkinje cells *in vitro*. Moreover, using two-photon measurements of GCaMP6f-encoded climbing fiber signals in Purkinje cells of intact mice, we observe enhanced responses to air puff stimuli applied to the whisker field. These findings resemble our previous phenotypic observations in a mouse model for the human 15q11-13 duplication, which does not extend to the *Cyfip1* locus. Thus, our study demonstrates that CYFIP1 overexpression shares a limited set of olivo-cerebellar phenotypes as those resulting from an increased number of copies of non-overlapping genes located on chromosome 15q11-13.

## Introduction

**FIGURE 1:**
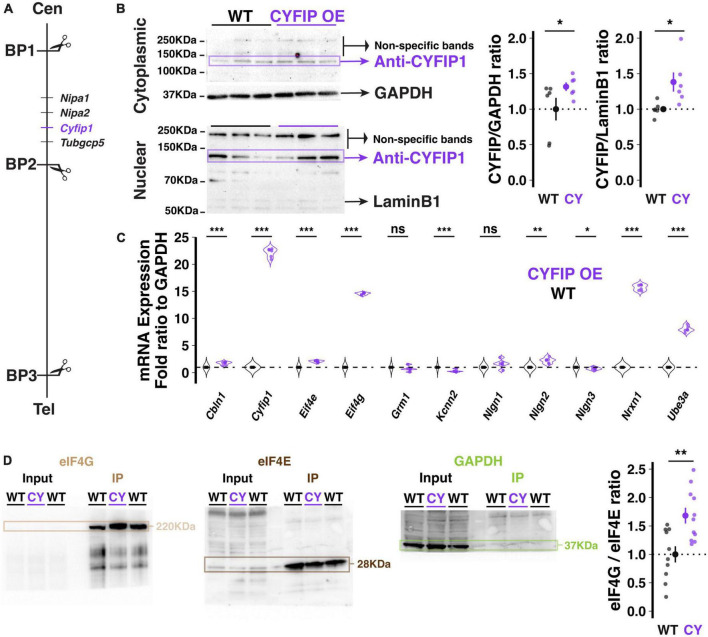
Genetic and molecular description of the CYFIP1 OE model. **(A)** Schematic of the location of the *Cyfip1* gene between BP1 and 2 of the murine chromosome 7. Cen: centromere; Tel: telomere. **(B)** Western blot demonstrates increased CYFIP1 protein in cerebellum of adult CYFIP1 OE mice *in cytoplasmic* (left, top) and nuclear fractions (left, bottom). Relative overexpression to WT mean across cytoplasmic and nuclear fractions (right). **(C)** The mRNA expression profiles of several candidate genes in CYFIPOE mice normalized to WT. **(D)** CYFIP1 OE mice have an elevated eIF4G/eIF4E binding ratio as determined by co-immunoprecipitation via m^7^GTP bead pull-down assay and western blot quantification. For each sample, eIF4E and eIF4G were probed on the same membrane that was then cut at ∼70 KDa for visualization. GAPDH expression was measured in input and pull-down to confirm specificity of the assay. The protein ratios are normalized to the WT group average. For panels **(B,D)**, large inset points indicate mean ± SEM while smaller points depict individual values.

The proximal long arm of human chromosome 15 is a frequent hot spot for copy number variations due to the local existence of multiple chromosomal breakpoints (BPs). The Prader-Willi/Angelman syndrome deletion region (PWACR; 15q11.2-13.1) is flanked by BP1 or BP2 proximally and BP3 distally, with more severe symptoms resulting from a larger deletion (Butler et al., 2004; Hartley et al., 2005). Duplication of the PWACR region results in dup15q syndrome, which is also frequently associated with autism spectrum disorder (ASD; Cook et al., 1997; Cook and Scherer, 2008). The BP1–BP2 region houses four genes (*Nipa1*, *Nipa2*, *Cyfip1*, and *Tubgcp5*; Figure 1A; Chai et al., 2003). Microdeletion of this region (Burnside-Butler syndrome) as well as its microduplication can result in autism and developmental delays, including motor and language delays (Burnside et al., 2011). In a Dutch family study, it was found that of three individuals with a BP1–BP2 microduplication in a seven-member family, all exhibited autism symptoms (van der Zwaag et al., 2010), which highlights the need to study phenotypes related to enhanced dosages of the four genes located in this region−in particular *Nipa1/2* and *Cyfip1*, which are highly expressed in the brain.

In mouse models of autism, CYFIP1, as a binding partner of the Fragile X Messenger Ribonucleoprotein (FMRP; Schenck et al., 2001), has been studied in some detail. Notably, in *Cyfip1* heterozygous knockout mice some phenotypes related to hippocampal plasticity were observed that mimicked the phenotypic profile described in *Fmr1* knockout mice (Bozdagi et al., 2012). *Cyfip1* overexpression has also been shown to alter spine density and maturation in cultured mouse hippocampal neurons (Pathania et al., 2014; Davenport et al., 2019) and neocortical pyramidal neurons (Oguro-Ando et al., 2015). CYFIP1 is enriched at excitatory (Pathania et al., 2014) and inhibitory synapses and alterations of Cyfip1 dosage produce bi-directional changes in the balance of excitation and inhibition, with Cyfip1 overexpression enhancing excitatory and diminishing inhibitory synapses (Davenport et al., 2019). Moreover, an exaggerated and generalized fear response has been observed in fear conditioning in these mice, while no abnormalities were found in behavioral test batteries assessing ASD-typical social or repetitive behaviors (Fricano-Kugler et al., 2019). This is different from observations made in a mouse model for the human 15q11-13 duplication (patdp/+), in which a conserved linkage group of mouse chromosome 7−corresponding to the BP2–BP3 region on human chromosome 15q11-13−was duplicated using chromosomal engineering. These mice show abnormalities in social interactions, behavioral inflexibility and increased anxiety (Nakatani et al., 2009).

Cerebellar involvement in cognitive function has been suggested for decades (Cerebellar cognitive affective syndrome; Schmahmann and Sherman, 1998) and it has more recently been suggested that cerebellar dysfunction during a critical postnatal period may be one of the strongest predictive factors in ASD (Wang et al., 2014). With these developments in mind, we have previously analyzed cerebellar function and sensorimotor behaviors in the patdp/+ mouse model for the human 15q11-13 duplication described above (Nakatani et al., 2009). We found abnormalities in locomotion and associative learning in these mice (Piochon et al., 2014) as well as enhanced climbing fiber-mediated responses to sensory input in Purkinje cell dendrites that were associated with aversive behaviors (Simmons et al., 2022). Localization of the synaptic organizer molecule neurexin 1 (NRXN1) was heightened at climbing fiber input sites on the Purkinje cell primary dendrite and we found that climbing fiber excitatory post-synaptic currents (EPSCs)−recorded from Purkinje cells in cerebellar slices using the whole-cell patch-clamp technique−were increased in amplitude (Simmons et al., 2022).

In the present work, we examined cerebellar climbing fiber signaling in CYFIP1 overexpressing (CYFIP1 OE) mice (originally described in Oguro-Ando et al., 2015). We make the same key observations of enhanced climbing fiber signaling that we made in patdp/+ mice with normal *Cyfip1* gene dosage: (a) increased NRXN1 localization to climbing fiber terminals onto Purkinje cell dendrites, (b) increased amplitudes of CF-EPSCs, and (c) larger responses to sensory stimulation as measured using two-photon imaging of dendritic calcium signals in intact mice. Although only reported for the better studied deletions, and not the duplications, symptoms in human patients are more severe when the longer area BP1–BP3, rather than just BP2–BP3, undergoes a change in copy number. The phenotypic resemblance of CYFIP1 overexpression to the BP2–BP3 duplication demonstrates a potential pathway for an increase in symptom severity with the longer BP1–BP3 duplication.

## Results

Transgenic mice harboring a bacterial artificial chromosome (BAC) spanning the *Cyfip1* locus and previously shown to over-express CYFIP1 protein in frontal cortex (Oguro-Ando et al., 2015), also exhibited enhanced CYFIP1 protein in the cerebellum at a level that is significantly higher than that of wild-type littermates (Figure 1B; Cytoplasmic fraction at 1.32 ± 0.024, *p* = 0.0538; Nuclear fraction at 1.38 ± 0.055, *n* = 6 CYFIP1 OE and 6 WT mice). This observation validates CYFIP1 OE mice as a model for enhanced expression of CYFIP1 protein in the cerebellum.

To assess the potential effects of enhanced *Cyfip1* dosage on other molecular pathways, we used a targeted approach to determine Purkinje cell specific transcription profiles by performing laser capture microdissection (LCM) exclusively on the somata and proximal dendrites of Purkinje cells. The content of messenger RNA (mRNA) was quantified using real time PCR (qRT-PCR) after reverse transcription for selected candidate genes that we previously assessed in the patdp/+ mouse model of the human 15q11-13 duplication (Simmons et al., 2022). In our quantification, we focused on the synaptic organizer molecules neuroligin 1–3 (*Nlgn1*, *Nlgn2*, *Nlgn3*), cerebellin 1 (*Cbln1*), and neurexin 1 (*Nrxn1*); Ube3a (*Ube3a*); the translation control factors eIF4E (*Eif4e*), eIF4G (*Eif4g*), and CYFIP1 itself (*Cyfip1*), as well as SK2-type K^+^ channels (*Kcnn2*) and type 1 metabotropic glutamate receptors (mGluR1, *Grm1*). We observed alterations in many of these candidates (Figure 1C) but will highlight a few notable findings. First, *Cyfip1* mRNA is upregulated in CYFIP1 OE mice (22.17 ± 0.33-fold above WT; here and below, *p* < 0.001, *n* = 6 and 6 animals), consistent with the desired genotype. The discrepancy between mRNA and protein upregulation that we observe (2217 vs. 30–40%, respectively) is similar to that described in human patients with 15q11-13 duplications (∼2400 vs. ∼250%, respectively; Oguro-Ando et al., 2015). Note that those results were found in homogenized superior frontal gyrus tissue, which could explain the remaining difference in protein upregulation in homogenized cerebellar tissue that we present here. Second, *Eif4g* mRNA is upregulated (14.58 ± 0.08-fold above WT), which may constitute a homeostatic regulation in response to enhanced CYFIP1 expression. Third, *Nrxn1* mRNA is upregulated (15.7 ± 0.22-fold above WT). The latter observation is of interest as it recapitulates a finding that we previously made in patdp/+ mice, which led to our investigation of enhanced NRXN1 localization at climbing fiber-Purkinje cell synapses and enhanced climbing fiber transmission (Simmons et al., 2022).

As the FMRP/CYFIP1 complex regulates mRNA translation via its control of the formation of the eIF4G/eIF4E complex (Napoli et al., 2008; Santini et al., 2017), we used an m^7^GTP bead pull-down assay of translation complexes followed by western blot analysis to measure the eIF4G/eIF4E binding ratio in the cerebellum of CYFIP1 OE mice and wild-type littermates. Unexpectedly, we observed a significant increase in the eIF4G/eIF4E binding ratio in CYFIP1 OE mice (Figure 1D; 1.68 ± 0.14, *p* = 0.003, *n* = 11 CYFIP1 OE and 10 WT mice), likely due to the increase in eIF4G expression. Regardless, this finding indicates changes in translation profiles for proteins of interest in the CYFIP1 OE mice. In the following, we will focus on localization properties of the cell adhesion protein NRXN1.

**FIGURE 2:**
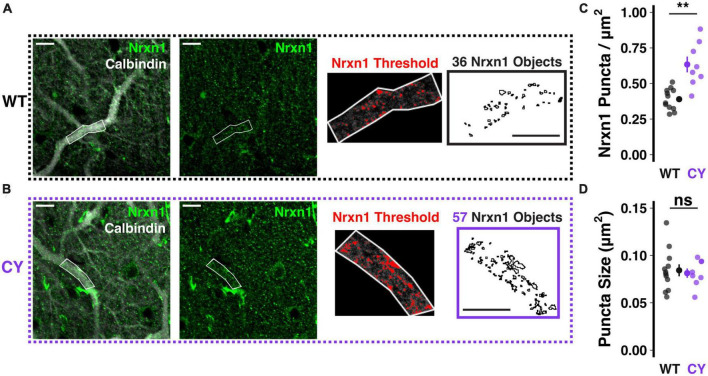
*Enhanced NRXN1* localization at putative CF terminals in CYFIP1 OE mice. **(A,B)** Immunohistochemical labeling reveals increased NRXN1 puncta along primary dendrites of calbindin-labeled Purkinje cells in CYFIP1 OE mice. The right two panels show the threshold applied to extract NRXN1 specific objects exclusively within the dendritic ROI. **(C)** Quantification of NRXN1 puncta per square micron area of primary dendrite ROIs. **(D)** Quantification of the average size of NRXN1 puncta within dendrite ROIs. For panels **(C,D)**, large inset points indicate mean ± SEM while smaller points depict individual values. Scale bars are each 10 μm. **p < 0.01.

At climbing fiber synapses to both soma and primary dendrite compartments, NRXN1 acts as a synaptic organizer, strengthening connectivity by forming trans-synaptic cell adhesion complexes with neuroligins 1 or 3 (Zhang et al., 2015; Chen et al., 2017). To test whether NRXN1 localization to climbing fiber synapses is enhanced in CYFIP1 OE mice, we performed immunohistochemical staining using antibodies against NRXN1 and calbindin, a calcium binding protein expressed densely and specifically throughout the dendritic and somatic compartments of Purkinje cells. We restricted our analysis of *NRXN1* localization to proximal branches of the primary dendrite (Figures 2A, B), which are most selectively contacted by the climbing fiber input (Rossi et al., 1991). In this measure, we observed an increase in the number of NRXN1 puncta in CYFIP1 OE mice as compared to WT littermates (Figure 2C; 0.63 ± 0.06 vs. 0.39 ± 0.02 puncta/μm^2^, *p* = 0.003, *n* = 8 and 12 dendrites, 4 and 4 mice). We did not observe a genotype difference in the average size of NRXN1 puncta (Figure 2D; 0.081 ± 0.005 vs. 0.084 ± 0.006 μm^2^, *p* = 0.714), suggesting that CYFIP1 over-expression selectively influences the maintenance of surplus synapses but not the gross size of the synapse to an extent that could be measured with this technique. It is not clear if the combined findings of increased Nrxn1 transcripts and NRXN1 localization to primary dendrite synapses also relates to increased NRXN1 protein expression overall.

**FIGURE 3:**
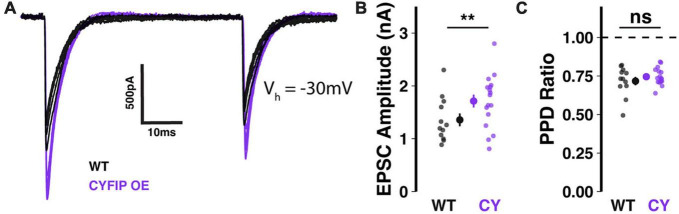
Enhanced CF-EPSCs in Purkinje cells of CYFIP1 OE mice **(A)** sample traces of stimulated CF-EPSCs recorded from WT and CYFIP1 OE Purkinje cells. Holding potential: –30 mV. **(B)** Quantification of CF-EPSC amplitudes. **(C)** Quantification of CF-EPSC paired pulse depression (EPSC2/EPSC1) with a 100 ms inter-stimulus interval. For panels **(B,C)**, large inset points indicate mean ± SEM while smaller points depict individual values. **p < 0.01.

In patdp/+ mice, enhanced NRXN1 localization at climbing fiber synapses may result in strengthened synaptic transmission (Simmons et al., 2022). To test whether the same relationship would be observed in CYFIP1 OE mice, we performed *in vitro* whole-cell patch-clamp recordings of climbing fiber-evoked excitatory post-synaptic currents (CF-EPSCs) in Purkinje cells filled with Cesium-based internal solution, to reduce space-clamp error, and kept at a −30 mV holding potential. Indeed, we found that CF-EPSC amplitudes were significantly larger when recorded from Purkinje cells in CYFIP1 OE mice than from Purkinje cells in WT littermates (Figures 3A, B; 1.71 ± 0.12 vs. 1.36 ± 0.12 nA, *p* = 0.048, *n* = 17 and 12 cells, 7 and 6 mice). On the other hand, CF-EPSC paired pulse depression (the ratio of EPSC2/EPSC1 during paired stimuli) was unaffected (Figure 3C; 0.72 ± 0.03 vs. 0.75 ± 0.01, *p* = 0.36, *n* = 17 and 12 cells, 7 and 6 mice), indicating that there is no genotype difference in pre-synaptic release probability.

**FIGURE 4:**
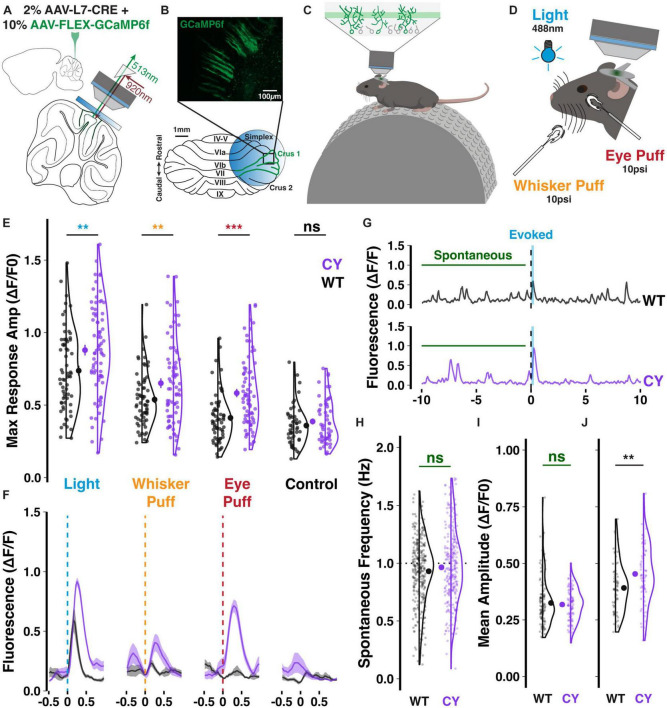
Enhanced sensory responsiveness of CYFIP1 OE crus 1 Purkinje cells *in vivo.* Schematics of panel **(A)** the dual injection of viral constructs and placement of an implanted cranial window; panel **(B)** the imaging configuration over lateral crus 1 and a sample field of view showing the sparse cellular label enabling calcium events from individual Purkinje cells to be separately deconvolved for analysis; panel **(C)** the experimental arrangement with mice head-restraining and resting on a treadmill during imaging of the intermediate Purkinje dendrites; and panel **(D)** the multisensory stimuli. **(E)** Purkinje cells of CYFIP1 OE mice exhibit larger calcium responses on average across sensory stimuli, but not during control trials without stimulus where any events during the response window are spontaneous (non-evoked) events. **(F)** Inter-trial calcium traces (mean with shaded SEM) of two individual cells, each exemplifying average WT and CYFIP1 OE responses. Given the mixed multi-sensory responsiveness of these cells, not all cells respond to every stimulus type equally, resulting in the heterogeneity of response size. **(G)** Sample traces from a 20 s trial of light stimulation exemplifying typical light-evoked responses and spontaneous events. Spontaneous events taken for quantification were sampled from the pre-stimulus period of each trial. **(H)** No difference in the spontaneous calcium event frequency between Purkinje cells of CYFIP1 OE and WT mice. **(I)** The mean amplitude of spontaneous calcium events is not different between genotypes. **(J)** The mean evoked calcium response–averaged over all sensory modalities–is larger in Purkinje cells of CYFIP1 OE mice. For panels **(E, H–J)**, large inset points indicate mean ± SEM while smaller points depict individual values. **p < 0.01, ***p < 0.001.

We next asked whether the abnormally strong climbing fiber input observed *in vitro* would translate into enhanced olivocerebellar signaling in the intact brain. To answer this, we dual injected adenovirus constructs encoding cre-recombinase on the Purkinje cell-specific L7 promoter (0.2 × 10^12^ GC/mL) and cre-dependent GCaMP6f (1 × 10^12^ GC/mL), a genetically encoded calcium indicator, to obtain specific expression in Purkinje cell dendrites of lateral crus 1 (Figures 4A, B). Lateral crus 1 is a region of the cerebellar hemisphere that is sensitive to multiple sensory modalities (Bosman et al., 2010; Ju et al., 2019) and which exhibits enhanced responsiveness to sensory stimuli in patdp/+ mice (Simmons et al., 2022). As GCaMP6f expression stabilized, mice were partially habituated to head-restraint over a treadmill (Figure 4C) and exposed to 30 ms sensory stimuli administered ipsilateral to the injected hemisphere, which receives climbing fiber input encoding ipsilateral receptive fields from the contralateral inferior olive. Stimuli included a 488 nm light and a 10 psi airpuff to the whisker pad (Figure 4D). As a benchmark, we also tested airpuffs delivered to the eye, a common unconditioned stimulus used for eye-blink conditioning and known to evoke climbing fiber-dependent complex spikes in Purkinje cells.

Sensory stimuli consistently evoked dendritic calcium events in GCaMP6f-expressing crus 1 Purkinje cell dendrites (Supplementary Figure 1). The maximum amplitude of sensory responses was larger in Purkinje dendrites of CYFIP1 OE mice than in those from WT littermates (Figures 4E, F). We observed elevated responses to Light (0.89 ± 0.04 vs. 0.74 ± 0.03 ΔF/F0, *p* = 0.005, *n* = 75 and 66 cells; here and below, cells are from 5 CYFIP1 OE and 5 WT animals; see Supplementary Figure 2), Whisker puff (0.65 ± 0.03 vs. 0.54 ± 0.02 ΔF/F0, *p* = 0.006, *n* = 73 and 65 cells), and Eye puff (0.58 ± 0.03 vs. 0.41 ± 0.02 ΔF/F0, *p* < 0.001, *n* = 74 and 65 cells), resembling the heightened over-responsivity across sensory modalities of patdp/+ mice (Simmons et al., 2022). Occasional non-evoked events during the response window of control trials−absent any stimulation−had lower maximum amplitudes than when a stimulus was presented and were equivalent between genotypes (Figure 4E; 0.41 ± 0.03 vs. 0.36 ± 0.02 ΔF/F0, *p* = 0.163, *n* = 54 and 49 cells).

Comparing pre-stimulus spontaneous events to evoked responses (Figure 4G), we did not observe a difference in the frequency of spontaneous events during the pre-stimulus periods of each trial (Figure 4H; 0.96 ± 0.02 vs. 0.93 ± 0.02 Hz, *p* = 0.171, *n* = 275 and 322 cells), but the typical 1 Hz frequency of both genotypes identifies these events as calcium transients caused by climbing fiber-evoked complex spikes (Simpson et al., 1996). Consistent with previous reports, the mean amplitude of spontaneous events is smaller than that of sensory-evoked ones (Figures 4I, J; Najafi et al., 2014). While the mean evoked amplitude is enhanced in CYFIP1 OE animals (Figure 4J; 0.45 ± 0.02 vs. 0.39 ± 0.01 ΔF/F0, *p* = 0.003, *n* = 75 and 66 cells), we did not observe a difference in the mean amplitude of spontaneous events between CYFIP1 OE and WT animals (Figure 4I; 0.32 ± 0.01 vs. 0.33 ± 0.01 ΔF/F0, *p* = 0.554, *n* = 96 and 109 cells).

**FIGURE 5:**
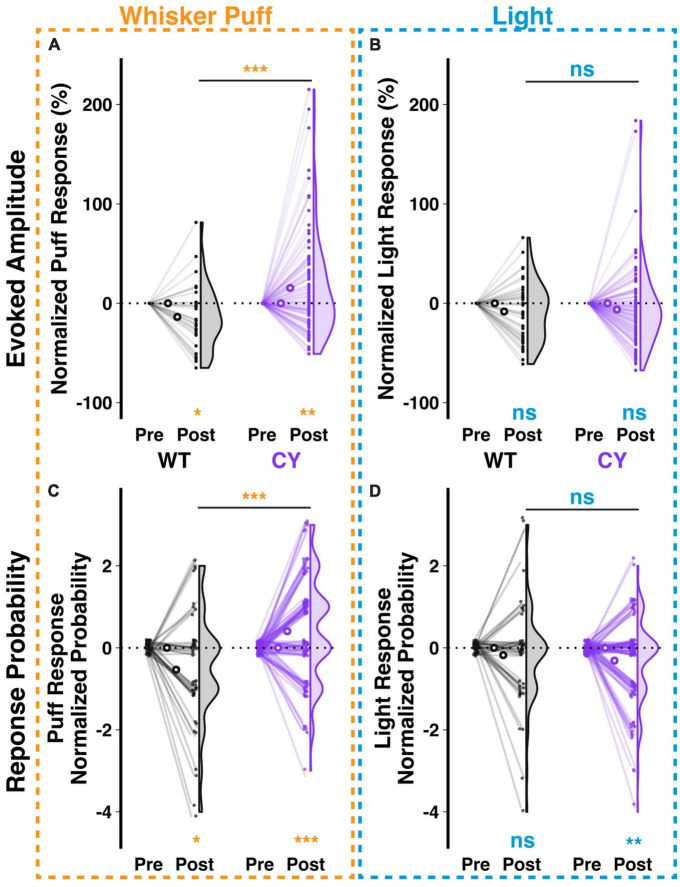
Repeated sensory exposure decreases responsiveness of WT cells but enhances that of CYFIP1 OE cells *in vivo*. **(A)** A repeated exposure paradigm (1 Hz whisker puffs for 5 min) induces opposite changes in response amplitude to subsequent whisker puff trials in Purkinje cells of WT vs. CYFIP1 OE mice. Here and below, normalized response is calculated as (Post – Pre)/Pre of ΔF/F0 or Post – Pre of the probability of response. **(B)** The same repeated whisker puff exposure paradigm has no effect on subsequent responses to light stimuli. **(C,D)** As in panels **(A,B)**, but for the calcium response probability. Panel **(C)** There is a decrease in the whisker puff response probability of WT cells and an increase in the response probability of CYFIP1 OE cells following repeated exposure to whisker puff. Panel **(D)** The same repeated whisker puff exposure has no effect on subsequent response probability to light stimuli. For panels **(A–D)**, large inset, hollow points indicate mean ± SEM while smaller points depict individual values.

Repeated stimulus presentation may have an impact on response amplitudes. In the case of the climbing fiber mediated responses studied here, we observed in WT animals that repeated exposure to a whisker puff (1 Hz for 5 min) reduced the amplitude (−13.72 ± 5.04%, *p* = 0.028, *n* = 42 cells from 5 animals) and probability (−0.53 ± 0.21 responses out of 5 trials per stimulus type during the first 20 min of exposure, *p* = 0.016, *n* = 45 cells) of responses to subsequent test pulses of whisker puff (raw and normalized changes shown in Supplementary Figures 3A, C and Figures 5A, C respectively). In contrast, Purkinje cells of CYFIP OE mice exhibited enhanced response amplitude (15.41 ± 5.05%, *p* = 0.005, *n* = 106 cells from five mice) and probability (0.41 ± 0.12 responses, *p* = 0.002, *n* = 106 cells) to whisker puff following repeated exposure (Figures 5A, C). As a control, repeated exposure to whisker puffs did not have a genotype specific effect on Purkinje cell responsiveness to light stimuli in either WT or CYFIP1 OE animals, though the response probability decreased modestly in cells from CYFIP1 OE mice (Supplementary Figures 3B, D and Figures 5B, D−0.31 ± 0.1 responses, *p* = 0.003, *n* = 106 cells). Genotype differences in untrained multisensory responses are easily obscured at the level of the averaged population response in each animal (Supplementary Figure 2); however, a consistent repeated exposure protocol, and the subsequent analysis of normalized change instead of absolute response amplitude, can provide a better assessment of genotype differences at the level of mice as well as cells. Indeed, the effects of repeated exposure could also be observed when averaging cells in each mouse (Supplementary Figure 4).

Cerebellar lateral crus 1 is responsive to multiple stimulus modalities, and many –but not all– individual cells are themselves responsive to all modalities (Ju et al., 2019). While the previous analysis identified trends in the whole population of WT and CYFIP1 OE cells, we next analyzed subsets of cells based on their stimulus responsiveness to assess the contributions of different subgroups to the trends in the population (Supplementary Figure 5). We observed that changes in population responsiveness were a factor both of changes in the number of cells with at least moderate response frequency (a decrease among WT cells and an increase among CYFIP OE cells; Supplementary Figures 5A, B, D, E) and changes in response frequency among cells that were consistently at least moderately responsive before and after the repeated exposure (Supplementary Figures 5C, F).

## Discussion

The main finding presented here is that phenotypes in olivocerebellar signaling are similar between CYFIP1 OE mice and the mouse model for the human 15q11-13 duplication. This notion holds for enhanced NRXN1 localization on the primary dendrite, enhanced CF-EPSC amplitudes as well as enhanced climbing fiber-mediated calcium events recorded from Purkinje cells in awake mice. All of these phenotypes were previously reported in the mouse model for the human 15q11-13 duplication (Simmons et al., 2022) and are in keeping with findings, albeit in hippocampus, that increased Cyfip1 dosage enhances excitatory synapses (Davenport et al., 2019). Intriguingly, increased excitatory synaptic density and size is particularly pronounced on the dendritic shaft of pyramidal neurons, where greater space may permit more expansion of synaptic area, which is the same region of Purkinje cell dendrites that is predominantly innervated by CF terminals with variable sizes of synaptic densities (Xu-Friedman et al., 2001).

The molecular reason for the phenotypic resemblance of patdp/+ and CYFIP1OE mice is currently not known. However, our finding speaks to the observation that clinical symptoms in patients with a long BP1–BP3 copy number variation are often more severe than symptoms in patients with a shorter BP2–BP3 one (Butler et al., 2004; Hartley et al., 2005). Although reported for deletions, the phenotype resemblance demonstrated here provides a proof-of-principle example how the same phenotypes might have their origin in BP1–BP2 as well as BP2–BP3, providing a basis for the added severity effect in BP1-3 alterations.

The consequences of pathologically enhanced signaling in the olivocerebellar system for patients with ASD are potentially manifold. One is that climbing fiber responses function as error signals in sensory-motor signaling (Marr-Albus-Ito models; see Ito, 2001). Abnormally strong climbing fiber responses to sensory stimuli might contribute to sensory defensiveness in autistic children, which includes avoidance behaviors to touch and other sensory stimuli (Markram et al., 2007). Moreover, climbing fiber co-activation promotes associative plasticity at parallel fiber to Purkinje cell synapses (Ito et al., 1982; Titley et al., 2019) and thus, ultimately, associative behavioral learning. Thus, too strong climbing fiber activity may abnormally promote associative plasticity, but it may also hinder it when preventing the proper development of parallel fiber input (as observed in patdp/+ mice; Piochon et al., 2014). In either case, pathologically enhanced climbing fiber activity will interfere with appropriate learning of associative relationships (Hansel, 2019).

An interesting observation made here is that repeated whisker stimulation at 1 Hz for 5 min causes a reduction in sensory-evoked calcium events in awake WT mice. A reminiscent effect has been described in the visual system and is known as “repetition suppression” (Desimone, 1996; Henson and Rugg, 2003). The reduction observed here outlasts the whisker stimulation and therefore includes a plasticity component. As this effect appears modality-specific−responses to whisker, but not light stimuli are significantly reduced−it is possible that it reflects long-term depression (LTD) of a parallel fiber component that is co-activated with the climbing fiber (Najafi et al., 2014). An interesting, alternative possibility is that this reduction reflects LTD at the climbing fiber input itself (Hansel and Linden, 2000; Carta et al., 2006), which can be monitored as a depression of widespread, dendritic calcium transients (Weber et al., 2003) and might show at different efficiencies when test responses are differently measured (same climbing fiber, but response to different sensory stimuli). CF-LTD as well as a transient reduction in climbing fiber transmission is promoted by the release of the neuropeptide corticotropin-releasing factor (CRF) from climbing fiber terminals and subsequent PKA/PKC activation in Purkinje cells (Schmolesky et al., 2007). Whatever the mechanism, the fact that in CYFIP1 OE mice repeated climbing fiber activation does not lead to any transient or lasting response reduction adds to the list of abnormalities in the olivocerebellar system described here.

Abnormal climbing fiber connectivity and/or transmission strength has also been found in motor diseases that involve the cerebellum, such as essential tremor and spino-cerebellar ataxia type 6 (Lin et al., 2014; Du et al., 2019). This observation shows how sensitive proper cerebellar function is to alterations in climbing fiber signal strength. The particular phenotypes resulting from these alterations will depend on variations in cerebellar regions affected as well as on a host of accompanying abnormalities.

## Materials and methods

### Animals

All animal experiments were approved and carried out in accordance with the regulations and guidelines for the care and use of experimental animals from the Institutional Animal Care and Use Committee of The University of Chicago (according to National Institute of Health guidelines).

### Statistics and quantifications

Animals of both sexes were used across experiments and no differences were observed. Statistical analysis was carried out using R (v4.2.1). Data following a normal distribution was tested with unpaired two-tailed Student’s *t*-tests to compare two groups. A one sample Student’s *t*-test was used to compare individual groups with a specific, benchmark value where appropriate. For all analyses, α = 0.05 was used to determine significance and figure panels refer to the significance of comparisons in the following way: ns *p* > 0.05, *p ≤ 0.05, ^**^p ≤ 0.01, ^***^p ≤ 0.001.

### Western blots

Western blot analysis was performed as previously described (Du et al., 2019): cerebellar tissue was collected from 2 to 4 months old mice. Nuclear and cytoplasmic protein fractions were then extracted by homogenization in NE-PER Nuclear and Cytoplasmic Extraction Reagents (Thermo Fisher Scientific, Cat# 78833). The lysates were quantified for protein concentration using the Bio-Rad Protein Assay Dye Reagent Concentrate (Cat# 5000006). Lysates were then diluted to normalize protein concentration and denatured in boiling water for 5 min. Then, 60 μg of protein was loaded on 8% SDS-Polyacrylamid gels in Tris-Glycine buffer. Gels were blotted on PVDF membranes (Millipore Sigma, Cat# IPVH00010) and blocked for 1 h in TBST containing 5% non-fat milk. Membranes were then incubated with primary antibodies (rabbit anti-PIR121/Sra-1 (CYFIP1, 1:500; Millipore, Cat# 07-531) and either mouse anti-GAPDH (1:20000; Thermofisher, Cat# AM4300) or mouse anti-LaminB1 (1:500; Santa Cruz, Cat# SC-374015) overnight at 4°C in TBST containing 5% non-fat milk. Membranes were then probed with HRP conjugated mouse IgG binding protein (1:20,000 and 1:2,000, for GAPDH and LaminB1, respectively; Santa Cruz, Cat# SC-516102) and mouse anti-rabbit (1:2,000; Santa Cruz, Cat# SC-2357) for 1 h at room temperature. Membranes were washed in TBST three times and then visualized with chemiluminescent detection using Clarity Western ECL Substrate (Bio-Rad, Cat# 170-5061). Images were captured using autoradiography or a ChemiDoc MP imaging system (Bio-Rad). Exported images were analyzed for densitometric quantification of bands by ImageJ (NIH). The CYFIP1/GAPDH and CYFIP1/LaminB1 values for each animal were normalized to the WT mean.

### Pull down co-immunoprecipitation and western blot analysis

Pull-down assays were performed as previously described (Santini et al., 2017). Mouse cerebellar tissue was collected after carbon dioxide anesthesia and was snap-frozen in dry ice. Tissues were sonicated in cold lysis buffer containing 150 mM NaCl, 10 mM MgCl_2_, 30 mM tris buffer (pH 8.0), 1 mM DTT, 1.5% Triton X-100, protease and ribonuclease inhibitors (10 μl/ml). A total of 500 μg of lysate were incubated with 30 μl of m^7^GTP beads (#AC155; Jena Bioscience) for 1 h at 4°C. The beads were centrifuged for 1 min at 6000 rpm, and the supernatant was collected. The beads were then washed three times in wash buffer containing 100 mM KCl, 50 mM tris buffer (pH 7.4), 5 mM MgCl_2_, 0.5% Triton X-100. Finally, the beads were eluted with 5X Laemmli buffer and analyzed using western blotting. The following antibodies were used in the western blotting analysis: rabbit anti-eIF4E (1:1000; Bethyl Laboratories; Cat# A301-153A) and rabbit anti-eIF4G (1:1000; Cell Signaling Technology; #C45A4). eIF4E and eIF4G were probed on the same membrane for each sample and the gel was then cut ∼70 KDa for visualization. Quantification is expressed as a ratio of eIF4G/eIF4E in each sample normalized to the average across WT controls such that the WT mean binding ratio is 1 and that of CYFIPOE animals is 1.68 times higher. Protein quantification was carried out before pull-down and loaded in equal volume for western blotting analysis. The assay was checked for specificity by measuring GAPDH expression with rabbit monoclonal antibodies (1:1,000; Cell Signaling Technology; Cat# 2118) in the input and pull-down.

Samples were prepared with 5X sample buffer (0.25 M Tris–HCl pH6.8, 10% SDS, 0.05% bromophenol blue, 50% glycerol and 25%–β mercaptoethanol) and heat denatured at 95°C for 5 min. A total of 40 μg protein per lane was run in pre-cast 4–12% Bis1053 Tris gels (Invitrogen) and subjected to SDSPAGE followed by wet gel transfer to polyvinylidene difluoride (PVDF; Immobilon-Psq, Millipore Corporation, Billerica, MA, USA) membranes. Membranes were blocked for 90 min with 5% milk in Tris-buffered saline supplemented with 0.1% Tween-20 (TBST) and then were probed overnight at 4°C using rabbit anti-eIF4E (1:1000; Bethyl Laboratories; Cat# A301-153A) and rabbit anti-eIF4G (1:1000; Cell Signaling Technology; #C45A4). Membranes were probed with horseradish peroxidase-conjugated secondary IgG (1:7000; Promega) for 1 h at room temperature. Signals from membranes were detected with ECL chemiluminescence (GE Healthcare Amersham™) using Alpha Imager 3.4 software and the FluorChem Protein Simple instrument and quantified via densitometry using ImageJ software (NIH, Bethesda, MA, USA).

### Laser-capture microdissection (LCM) of PCs, mRNA isolation, and quantitative real-time PCR

Laser-capture microdissection and mRNA isolation were performed as previously reported (Du et al., 2019). Frozen cerebellar sections (10 μm thick; ∼P100 mice) were cut on a Cryostat NX50 (Leica, Milton Keynes, UK). Sections were attached to RNase free PEN-membrane slides (Leica, Milton Keynes, UK) and stained with fast HE staining (ScyTek Labs, UT, USA). A total of 30 Purkinje cells (somatic and proximal dendritic tissue) were isolated from each side of the cerebellum in three slices per animal, for each group (360 Purkinje cells and six RNA samples per genotype; two mice per group) and collected in the tube using Leica LMD 6500 for subsequent RNA isolation. Total RNAs were extracted using Arcturus Picopure RNA isolation Kit (Thermo Fisher, VA, USA). All samples passed Bioanalyzer RNA QC analysis (Agilent, CA, USA), with a RNA integrity Number (RIN) of at least 7. Each 25 μL reaction solution contained 2 μL primers, 12.5 μL SYBR green Supermix (BioRad, CA, USA) and was run under the following conditions: 50°C 2 min; 95°C 10 min; 95°C 15 s, 60°C 1 min for 40 cycles, followed by a melting curve. As a relative quantification, fold changes were measured using the ΔΔCt method, with *Gapdh* as an internal control.

### Immunohistochemistry

Experiments were performed on CYFIP OE and WT mice aged 13–15 weeks. Mice were anesthetized with sterile deionized water containing 10% ketamine (Henry Schein) and 5% xylazine (Akorn), then perfused with 4% paraformaldehyde (PFA). Cerebella were removed, incubated overnight in 4% PFA at 4°C, incubated for 24 h in 30% sucrose solution at 4°C, and then sliced (50 μm, sagittal) using a cryostat microtome (CM 3050S, Leica). Permeabilization was done in deionized water containing 0.025% triton (TX) and 10% phosphate buffered saline (PBS) for 1 h at room temperature (RT). Blocking was done with PBS-TX containing 10% normal donkey serum for 2 h at RT. The primary antibody incubation took place overnight at 4°C with a 1% normal donkey serum in PBS-TX solution containing: mouse anti-NRXN1 (1:200; Millipore; Cat# MABN607) and guinea pig anti-calbindin (1:1000; Synaptic Systems; Cat# 214 004; RRID:AB_10550535). After 3 × 10 min washes in PBS-TX at RT, the secondary antibody incubation lasted for 2 h at 4°C in a 1% normal donkey serum in PBS-TX solution containing: donkey anti-guinea pig, CY3 (1:200, Jackson ImmunoResearch; Cat# 711-165-152; RRID:AB_2307443), and donkey anti-mouse, AF488 (1:200, Jackson ImmunoResearch; Cat# 715-545-150; RRID:AB_2340846). Following antibody binding, slices were washed PBS-TX for 3 × 10 min, mounted and coverslipped with Vectashield (Vector Laboratories, Inc.), and allowed to dry overnight before visualization.

Slices were imaged at 40x (Apochromat 1.3NA, oil immersion) and z-stacks of the molecular layer were obtained (11 images with a 0.431 μm z-step for a total height of 4.89 μm) with a confocal microscope (Zeiss LSM 5 Exciter, Axioskop 2). Separate images, 75 μm × 75 μm × 4.89 μm, were collected from proximal portions of the molecular layer in Lobules 3–5 of 4 stained sections per animal.

Image processing was performed using ImageJ (NIH) on blinded images. Image channels used for analyses were separated and thresholded using the Triangle threshold operation (Zack et al., 1977). To count NRXN1 punctae on proximal dendritic segments (Figure 2), a 100 μm^2^ ROI was traced over large caliber dendrites visible in the central plane (image 6 of 11) of the calbindin channel z-stack. This ROI was used as a mask to obtain counts using the 2D Analyze Particles function (ImageJ) in the central plane of the NRXN1 channel. This method was repeated to get two 100 μm^2^ dendritic ROIs per animal from different images. Blinded image data was compiled in an excel database and subsequently a custom R script decoded animal ID, section number, and genotype from image numbers. Measurements are expressed as mean ± standard error of the mean (SEM).

### Slice preparation and electrophysiology

Young adult mice (P23-50) were anesthetized with isoflurane and decapitated. The cerebellar vermis was removed and cooled in artificial cerebrospinal fluid (ACSF) containing (in mM): 124 NaCl, 5 KCl, 1.25 Na2HPO_4_, 2 CaCl2, 2 MgSO_4_, 26 NaHCO_3_, and 10 D-glucose, bubbled with 95% O_2_ and 5% CO_2_. Sagittal slices of the cerebellar vermis (200 μm thick) were prepared using a Leica VT-1000S vibratome and were subsequently kept for at least 1 h at room temperature in oxygenated ACSF. For recordings, the slices were perfused with ACSF that was supplemented with picrotoxin (100 μM) to block GABA_A_ receptors.

Patch-clamp recordings from the Purkinje cell soma were performed at room temperature using an EPC-10 amplifier (HEKA Electronics). Currents were filtered at 3 kHz, digitized at 25 kHz, and acquired using Patchmaster software (HEKA Electronics). Patch pipettes (2–5 MΩ) were filled with a solution containing (in mM): 128 CsOH, 111 gluconic acid, 4 NaOH, 10 CsCl, 2 MgCl2, 10 HEPES, 4 Na2ATP, 0.4 Na3GTP, and 30 sucrose (osmolarity: 295–305 mmol/kg, pH 7.25). Cells were held at −30 mV holding potential. CF inputs were activated using glass pipettes filled with ACSF (0.2 ms stimulus duration with intensities ranging from 1–150 nA). Liquid junction potentials were not corrected. Fast and slow capacitances were compensated and series resistance was partially compensated (50–80%). Stimulus intensity and location were not systematically varied to search for multiple CF inputs. CF excitatory post-synaptic currents (EPSCs), particularly of reduced amplitude in some multi-innervated cells, were distinguished from parallel fiber (PF)-EPSCs by their paired pulse depression (100 ms interval, EPSC2/EPSC1) and stable amplitude with small changes in stimulus intensity. PF-EPSCs exhibit paired pulse facilitation and linear amplitude relationship with even small stimulus intensity changes.

### *In vivo* two-photon imaging from awake mice

Surgeries were performed as described previously (Simmons et al., 2022) on animals aged 11–13 weeks under ketamine/xylazine anesthesia (100 and 10 mg/kg, respectively, 0.1 mL/10 g weight; Henry Schein) with subcutaneous injections of meloxicam (0.06 mL, 1–2 mg/kg), buprenorphine (0.1 mL, 0.05–0.1 mg/kg), and sterile saline (1 mL). Body temperature was maintained at 35–37°C with a feedback dependent heating pad. The skin above the posterior skull was excised and the bone cleaned to implant a metal headframe over the interparietal bone via dental cement. After 1–4 days of recovery, mice were anesthetized and a 4 mm craniotomy and durectomy was made at 2.5 mm lateral from midline and 2.5 mm caudal from lambda, exposing cerebellar simplex, crus 1, and anterior crus 2. Combined low titer PC-specific L7-Cre (0.2 × 10^12^ GC/mL, AAV1.sL7.Cre.HA.WPRE.hGH.pA; Princeton Neuroscience Institute (PNI) Viral Core Facility; acquired from Dr. Samuel Wang Lab, Princeton University) and high titer Cre-dependent GCaMP6f (1 × 10^12^ GC/mL, pAAV.CAG.Flex.GCaMP6f.WPRE.SV40; Addgene plasmid # 100835) was injected ∼300 μm below the pial surface of central simplex and crus 1 (∼900 nL per site, 5 min wait before needle retraction) and a cranial window was implanted over the craniotomy.

The mice recovered for 5 days before daily habituation to the imaging apparatus, head restraint on the treadmill, and multisensory stimulus application (6–10 days) to encourage resting behavior to reduce the conflation of running motor programs on cerebellar activity. Animals were habituated until they exhibited relative comfort and reduced running behavior. Imaging experiments were performed when the GCaMP6f indicator reached stable expression in a sparse cell population (20–30 days post-injection). PC dendrites were imaged at 31 Hz using a laser scanning two-photon microscope (Neurolabware) and 16x water immersion objective (0.8 NA, 3 mm WD; Nikon). GCaMP6f was excited at 920 nm with a femtosecond-pulsed two- photon laser (∼30 mW laser power; Spectra-Physics) and fluorescence collected by a GaAsP PMT (Hamamatsu).

During each experiment, calcium activity was monitored in ∼15–25 cells per animal during 20 s imaging sessions. One of three stimulus types (1. Light, 2. Airpuff (whisker), and –to include a stimulus that is well-known to evoke complex spikes– 3. Airpuff to the eye) was triggered 10 s after scanning initiation and lasted for 30 ms. Light stimulus was a 488 nm LED light (Prizmatix) targeted to the ipsilateral eye, Air Puff was delivered at 10 psi (Picospritzer III, Parker Hannifin) via a 0.86 mm diameter capillary tube positioned 2–3 mm from the center of the ipsilateral whisker pad or ipsilateral eye.

For repeated exposure experiments, stimulus trials were reduced to 10 s duration with stimuli delivered after a 5 s delay. Ten trials each of either light or whisker airpuff (five of each) and three control trials without stimulus were conducted before and after the repeated exposure paradigm. The repeated exposure paradigm consisted of 30 ms whisker airpuff delivered at 1 Hz for 5 min without any imaging sessions.

The stimuli were applied with inter-stimulus intervals ≥30 s. An Arduino Uno microcontroller triggered by the imaging software provided distinct stimulus type triggering output to the light and puff instruments. The microcontroller was programmed to cycle through stimulus types randomly and included control sessions–imaging under identical conditions but without any stimulus delivered–and was repeated until 10 trials were acquired of each type.

Images were converted to tiffs and motion corrected using custom MATLAB scripts. Cellular ROIs were drawn manually in ImageJ based on volumetric cell reconstructions. Another MATLAB script measured the pixel intensity of each ROI across frames and videos and saved the data as a mat file. An interactive MATLAB GUI was used to manually confirm detection quality and consistency across imaging sessions to either include or exclude each cell for downstream analysis. Analyses were performed using MATLAB scripts and output for final data shaping, plotting, and statistics in R.

Raw signal from all ROIs was imported to a custom MATLAB script that performed a five-frame moving window smoothing function and a background correction function. Raw ROI traces were input to the MATLAB version of OASIS deconvolution to obtain rise initiation times of calcium peaks exceeding 3SD of the baseline. Event amplitude was determined as the maximum value of the smoothed trace within 3 frames of the deconvolved times. Across animals, cells with saturated baseline fluorescence, determined by the baseline value at which spontaneous event amplitudes were consistently diminished, were removed. We did not distinguish between multiple tightly clustered events producing a single, accumulated large amplitude peak. While accumulated peaks from clustered inputs often retain multiple peaks (a partial peak within the rising phase of the larger event), the slow time constant of the GCaMP6f indicator can alter the appearance of multiple peaks and produce variable spike deconvolution. As such, we identified peak times <3 frames apart − having only 1 frame (31 ms) between detected peaks, which is below the ∼50 ms rise time constant of GCaMP6f (Chen et al., 2013) − and took only the second and highest peak or the last in a sequence of >2 events all of which are <3 frames apart.

While physiological responses to stimulation are expected to start as early as 50 ms after the stimulus, the GCaMP6f indicator provides a slower readout of stimulus response. Thus, calcium events were considered stimulus-evoked if the event rise initiation occurred within a time window of 225 ms (7 frames) that started 60 ms (2 frames) after the stimulus. For repeated exposure experiments, the trial average of the stimulus-evoked amplitude (measured as ΔF/F0 of trials including a response) is obtained for trials before (pre) and after (post) the repeated exposure protocol. Normalized change was calculated with the following formula: [(Post − Pre)/Pre] × 100. Response probability was calculated by subtracting the rate of evoked responses out of five trials Pre and Post (Post – Pre).

## Data availability statement

The original contributions presented in this study are included in this article/Supplementary material, further inquiries can be directed to the corresponding authors.

## Ethics statement

The animal study was reviewed and approved by the Institutional Animal Care and Use Committee of The University of Chicago.

## Author contributions

SB, CG, EK, and CH designed the experiments. SB, DS, EG, FL, and XD performed the experiments and analyzed the data. SB and CH wrote the manuscript. All authors contributed to the article and approved the submitted version.

## Supplementary material

Supplementary Figure 1 Sample raw traces across trials of light stimulus. **(A)** Responses to light stimulation presented with a 10 s delay during each of ten 20 s trials of stimulation from individual example WT and CYFIPOE cells. **(B)** Trial traces are overlaid for each cell in panel **(A)** to demonstrate the modest difference in average evoked amplitude.

Supplementary Figure 2 Multisensory responsiveness averaged by animal. **(A–D)** All cell data from Figures 4E, H–J averaged by animal. Large inset, hollow points indicate median while smaller points depict individual values.

Supplementary Figure 3 Un-normalized evoked Ca^2+^ amplitude and probability of response reflects the same effect of repeated exposure as observed with normalized data despite variable baseline responsiveness. **(A,B)** Raw Puff and Light evoked response ΔF/F0 values for each cell pre vs. post. **(C,D)** Raw Puff and Light evoked response probability for each cell out of 5 trials during the first 20 min before and after repeated exposure. For **(A–D)**, large inset, hollow points indicate mean ± SEM while smaller points depict individual values.

Supplementary Figure 4 Effects of repeated sensory exposure by animal. **(A–D)** As in Figures 5A–D, but with cellular data averaged across each animal. Large inset, hollow points indicate median while smaller points depict individual values.

Supplementary Figure 5 Genotype differences in population responsiveness are supported by changes across distinct types of cellular responsiveness. **(A)** More unresponsive CYFIPOE cells exhibit at least moderate responsiveness following repeated exposure than WT. Here, cells are defined as responsive if they exhibit more than one Ca^2+^ peak within the response window across five trials. This exceeds the ∼1/5 chance that ∼1 Hz spontaneous events may be present during a 250 ms response window. **(B)** Fewer responsive CYFIPOE cells exhibit a loss of detectable response than among WT cells. **(C)** Among cells that are consistently responsive, CYFIPOE cells show an increase in probability of response as compared with a decrease among WT cells. **(D–F)** As in panels **(A–C)** for Light stimuli where there is only a moderate effect of decreased responsiveness in the CYFIPOE cell population. For **(A–D)**, large inset, hollow points indicate mean ± SEM while smaller points depict individual values.

## References

[B1] Bosman L. Koekkoek S. Shapiro J. Rijken B. Zandstra F. van der Ende B. (2010). Encoding of whisker input by cerebellar Purkinje cells. *J. Physiol.* 588 3757–3783. 10.1113/jphysiol.2010.195180 20724365 PMC2998225

[B2] Bozdagi O. Sakurai T. Dorr N. Pilorge M. Takahashi N. Buxbaum J. (2012). Haploinsufficiency of Cyfip1 produces fragile X-like phenotypes in mice. *PLoS One* 7:e42422. 10.1371/journal.pone.0042422 PMC3416859 22900020

[B3] Burnside R. Pasion R. Mikhail F. Carroll A. Robin N. Youngs E. (2011). Microdeletion/microduplication of proximal 15q11.2 between BP1 and BP2: a susceptibility region for neurological dysfunction including developmental and language delay. *Hum. Genet.* 130 517–528. 10.1007/s00439-011-0970-4 21359847 PMC6814187

[B4] Butler M. Bittel D. Kibiryeva N. Talebizadeh Z. Thompson T. (2004). Behavioral differences among subjects with Prader-Willi syndrome and type I or type II deletion and maternal disomy. *Pediatrics* 113 565–573. 10.1542/peds.113.3.565 14993551 PMC6743499

[B5] Carta M. Mameli M. Valenzuela C. (2006). Alcohol potently modulates climbing fiber–>Purkinje neuron synapses: role of metabotropic glutamate receptors. *J. Neurosci.* 26 1906–1912. 10.1523/JNEUROSCI.4430-05.2006 16481422 PMC6674936

[B6] Chai J. Locke D. Greally J. Knoll J. Ohta T. Dunai J. (2003). Identification of four highly conserved genes between breakpoint hotspots BP1 and BP2 of the Prader-Willi/Angelman syndromes deletion region that have undergone evolutionary transposition mediated by flanking duplicons. *Am. J. Hum. Genet.* 73 898–925. 10.1086/378816 14508708 PMC1180611

[B7] Chen L. Jiang M. Zhang B. Gokce O. Südhof T. (2017). Conditional deletion of all neurexins defines diversity of essential synaptic organizer functions for neurexins. *Neuron* 94 611–625.e4. 10.1016/j.neuron.2017.04.011 28472659 PMC5501922

[B8] Chen T. Wardill T. Sun Y. Pulver S. Renninger S. Baohan A. (2013). Ultrasensitive fluorescent proteins for imaging neuronal activity. *Nature* 499 295–300. 10.1038/nature12354 23868258 PMC3777791

[B9] Cook E. Scherer S. (2008). Copy-number variations associated with neuropsychiatric conditions. *Nature* 455 919–923. 10.1038/nature07458 18923514

[B10] Cook E. H. Lindgren V. Leventhal B. Courchesne R. Lincoln A. Shulman C. (1997). Autism or atypical autism in maternally but not paternally derived proximal 15q duplication. *Am. J. Hum. Genet.* 60 928–934. 9106540 PMC1712464

[B11] Davenport E. Szulc B. Drew J. Taylor J. Morgan T. Higgs N. (2019). Autism and Schizophrenia-Associated CYFIP1 regulates the balance of synaptic excitation and inhibition. *Cell Rep.* 26 2037–2051.e6. 10.1016/j.celrep.2019.01.092 30784587 PMC6381785

[B12] Desimone R. (1996). Neural mechanisms for visual memory and their role in attention. *Proc. Natl. Acad. Sci. U. S. A.* 93 13494–13499. 10.1073/pnas.93.24.13494 8942962 PMC33636

[B13] Du X. Wei C. Hejazi Pastor D. Rao E. Li Y. Grasselli G. (2019). α1ACT Is Essential for survival and early cerebellar programming in a critical neonatal window. *Neuron* 102 770–785.e7. 10.1016/j.neuron.2019.02.036 30922876 PMC6533132

[B14] Fricano-Kugler C. Gordon A. Shin G. Gao K. Nguyen J. Berg J. (2019). CYFIP1 overexpression increases fear response in mice but does not affect social or repetitive behavioral phenotypes. *Mol. Autism* 10:25. 10.1186/s13229-019-0278-0 31198525 PMC6555997

[B15] Hansel C. (2019). Deregulation of synaptic plasticity in autism. *Neurosci. Lett.* 688 58–61. 10.1016/j.neulet.2018.02.003 29421544

[B16] Hansel C. Linden D. (2000). Long-term depression of the cerebellar climbing fiber–Purkinje neuron synapse. *Neuron* 26 473–482. 10.1016/s0896-6273(00)81179-4 10839365

[B17] Hartley S. Maclean W. Butler M. Zarcone J. Thompson T. (2005). Maladaptive behaviors and risk factors among the genetic subtypes of Prader-Willi syndrome. *Am. J. Med. Genet. A.* 136 140–145. 10.1002/ajmg.a.30771 15940679 PMC1896317

[B18] Henson R. Rugg M. (2003). Neural response suppression, haemodynamic repetition effects, and behavioural priming. *Neuropsychologia* 41 263–270. 10.1016/s0028-3932(02)00159-8 12457752

[B19] Ito M. (2001). Cerebellar long-term depression: characterization, signal transduction, and functional roles. *Physiol. Rev.* 81 1143–1195. 10.1152/physrev.2001.81.3.1143 11427694

[B20] Ito M. Sakurai M. Tongroach P. (1982). Climbing fibre induced depression of both mossy fibre responsiveness and glutamate sensitivity of cerebellar Purkinje cells. *J. Physiol.* 324 113–134. 10.1113/jphysiol.1982.sp014103 7097592 PMC1250696

[B21] Ju C. Bosman L. Hoogland T. Velauthapillai A. Murugesan P. Warnaar P. (2019). Neurons of the inferior olive respond to broad classes of sensory input while subject to homeostatic control. *J. Physiol.* 597 2483–2514. 10.1113/JP277413 30908629 PMC6487939

[B22] Lin C. Louis E. Faust P. Koeppen A. Vonsattel J. Kuo S. (2014). Abnormal climbing fibre-Purkinje cell synaptic connections in the essential tremor cerebellum. *Brain* 137 3149–3159. 10.1093/brain/awu281 25273997 PMC4240294

[B23] Markram H. Rinaldi T. Markram K. (2007). The intense world syndrome–an alternative hypothesis for autism. *Front. Neurosci.* 1:2007. 10.3389/neuro.01.1.1.006.2007 18982120 PMC2518049

[B24] Najafi F. Giovannucci A. Wang S. Medina J. (2014). Sensory-driven enhancement of calcium signals in individual Purkinje cell dendrites of awake mice. *Cell Rep.* 6 792–798. 10.1016/j.celrep.2014.02.001 24582958 PMC3996650

[B25] Nakatani J. Tamada K. Hatanaka F. Ise S. Ohta H. Inoue K. (2009). Abnormal behavior in a chromosome-engineered mouse model for human 15q11-13 duplication seen in autism. *Cell* 137 1235–1246. 10.1016/j.cell.2009.04.024 19563756 PMC3710970

[B26] Napoli I. Mercaldo V. Boyl P. Eleuteri B. Zalfa F. De Rubeis S. (2008). The fragile X syndrome protein represses activity-dependent translation through CYFIP1, a new 4E-BP. *Cell* 134 1042–1054. 10.1016/j.cell.2008.07.031 18805096

[B27] Oguro-Ando A. Rosensweig C. Herman E. Nishimura Y. Werling D. Bill B. (2015). Increased CYFIP1 dosage alters cellular and dendritic morphology and dysregulates mTOR. *Mol. Psychiatry* 20 1069–1078. 10.1038/mp.2014.124 25311365 PMC4409498

[B28] Pathania M. Davenport E. Muir J. Sheehan D. López-Doménech G. Kittler J. (2014). The autism and schizophrenia associated gene CYFIP1 is critical for the maintenance of dendritic complexity and the stabilization of mature spines. *Transl. Psychiatry* 4:e374. 10.1038/tp.2014.16 24667445 PMC3966042

[B29] Piochon C. Kloth A. Grasselli G. Titley H. Nakayama H. Hashimoto K. (2014). Cerebellar plasticity and motor learning deficits in a copy-number variation mouse model of autism. *Nat. Commun.* 5:5586. 10.1038/ncomms6586 25418414 PMC4243533

[B30] Rossi F. Wiklund L. van der Want J. Strata P. (1991). Reinnervation of cerebellar Purkinje cells by climbing fibres surviving a subtotal lesion of the inferior olive in the adult rat. I. Development of new collateral branches and terminal plexuses. *J. Comp. Neurol.* 308 513–535. 10.1002/cne.903080403 1865015

[B31] Santini E. Huynh T. Longo F. Koo S. Mojica E. D’Andrea L. (2017). Reducing eIF4E-eIF4G interactions restores the balance between protein synthesis and actin dynamics in fragile X syndrome model mice. *Sci. Signal* 10:eaan0665. 10.1126/scisignal.aan0665 29114037 PMC5858943

[B32] Schenck A. Bardoni B. Moro A. Bagni C. Mandel J. L. (2001). A highly conserved protein family interacting with the fragile X mental retardation protein (FMRP) and displaying selective interactions with FMRP-related proteins FXR1P and FXR2P. *Proc. Natl. Acad. Sci. U. S. A.* 98 8844–8849. 10.1073/pnas.151231598 11438699 PMC37523

[B33] Schmahmann J. Sherman J. (1998). The cerebellar cognitive affective syndrome. *Brain* 121 561–579. 10.1093/brain/121.4.561 9577385

[B34] Schmolesky M. De Ruiter M. De Zeeuw C. Hansel C. (2007). The neuropeptide corticotropin-releasing factor regulates excitatory transmission and plasticity at the climbing fibre-Purkinje cell synapse. *Eur. J. Neurosci.* 25 1460–1466. 10.1111/j.1460-9568.2007.05409.x 17425571

[B35] Simmons D. Busch S. Titley H. Grasselli G. Shih J. Du X. (2022). Sensory over-responsivity and aberrant plasticity in cerebellar cortex in a mouse model of syndromic Autism. *Biol. Psychiatry Glob. Open Sci.* 2 450–459. 10.1016/j.bpsgos.2021.09.004 36324646 PMC9616247

[B36] Simpson J. I. Wylie D. R. De Zeeuw C. I. (1996). On climbing fiber signals and their consequence(s). *Behav. Brain Sci.* 19 384–398.

[B37] Titley H. Kislin M. Simmons D. Wang S. Hansel C. (2019). Complex spike clusters and false-positive rejection in a cerebellar supervised learning rule. *J. Physiol.* 597 4387–4406. 10.1113/JP278502 31297821 PMC6697200

[B38] van der Zwaag B. Staal W. Hochstenbach R. Poot M. Spierenburg H. de Jonge M. (2010). A co-segregating microduplication of chromosome 15q11.2 pinpoints two risk genes for autism spectrum disorder. *Am. J. Med. Genet. B Neuropsychiatr. Genet.* 153B 960–966. 10.1002/ajmg.b.31055 20029941 PMC2933514

[B39] Wang S. Kloth A. Badura A. (2014). The cerebellum, sensitive periods, and autism. *Neuron* 83 518–532. 10.1016/j.neuron.2014.07.016 25102558 PMC4135479

[B40] Weber J. De Zeeuw C. Linden D. Hansel C. (2003). Long-term depression of climbing fiber-evoked calcium transients in Purkinje cell dendrites. *Proc. Natl. Acad. Sci. U. S. A.* 100 2878–2883. 10.1073/pnas.0536420100 12601151 PMC151434

[B41] Xu-Friedman M. Harris K. Regehr W. (2001). Three-dimensional comparison of ultrastructural characteristics at depressing and facilitating synapses onto cerebellar Purkinje cells. *J. Neurosci.* 21 6666–6672. 10.1523/JNEUROSCI.21-17-06666.2001 11517256 PMC6763067

[B42] Zack G. Rogers W. Latt S. (1977). Automatic measurement of sister chromatid exchange frequency. *J. Histochem. Cytochem*. 25, 741–753. 10.1177/25.7.70454 70454

[B43] Zhang B. Chen L. Liu X. Maxeiner S. Lee S. Gokce O. (2015). Neuroligins sculpt cerebellar purkinje-cell circuits by differential control of distinct classes of synapses. *Neuron* 87 781–796. 10.1016/j.neuron.2015.07.020 26291161 PMC4545494

