# Supplementary figures and images for: Overexpression of the autism candidate gene Cyfip1 pathologically enhances olivo-cerebellar signaling in mice
Source: Front Cell Neurosci. 2023 Jul 20;17:1219270. doi: 10.3389/fncel.2023.1219270 (PMC10399232; doi:10.3389/fncel.2023.1219270)

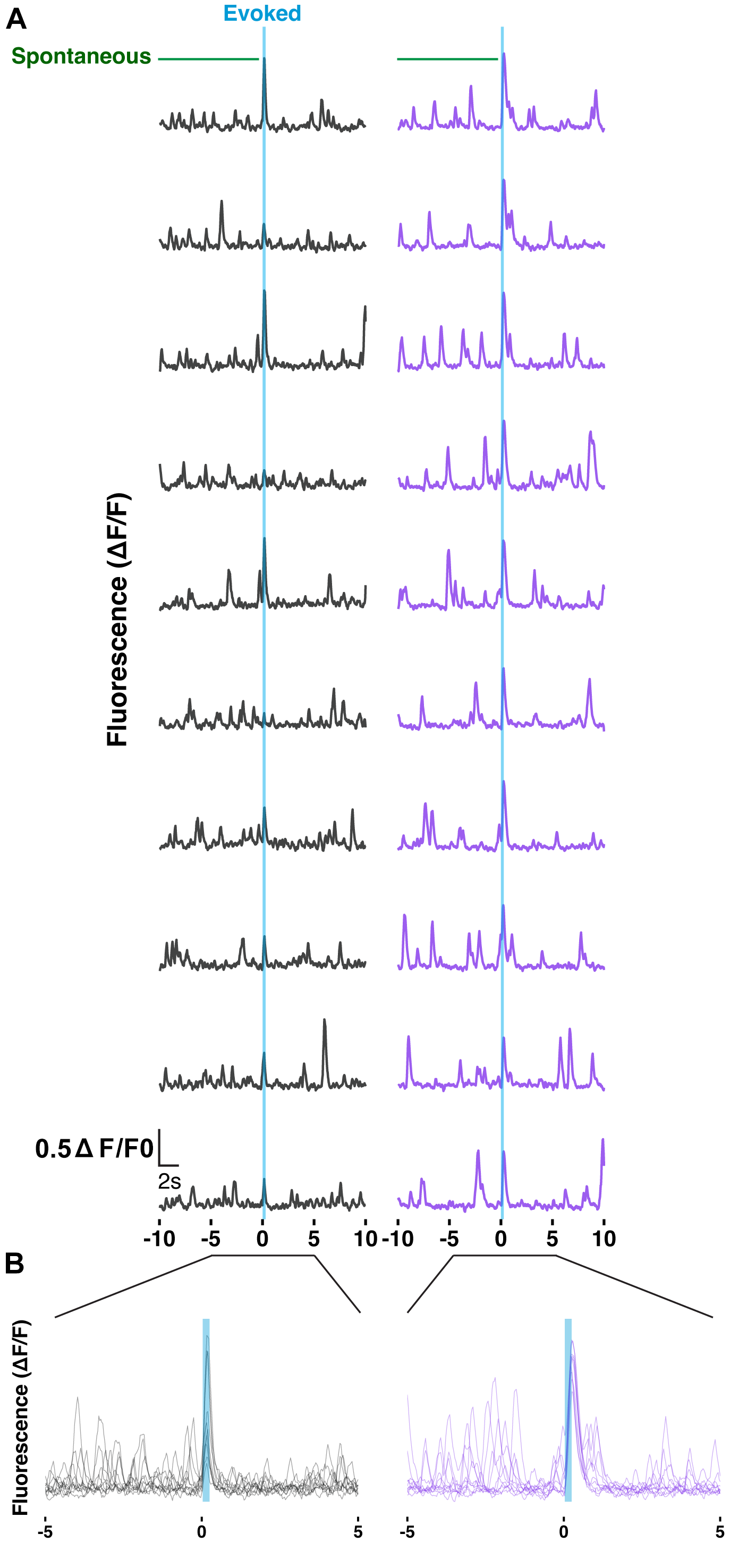

Supplement: Supplementary Figure 1 — Sample raw traces across trials of light stimulus. (A) Responses to light stimulation presented with a 10 s delay during each of ten 20 s trials of stimulation from individual example WT and CYFIPOE cells. (B) Trial traces are overlaid for each cell in panel (A) to demonstrate the modest difference in average evoked amplitude. [file Image_1.tif]

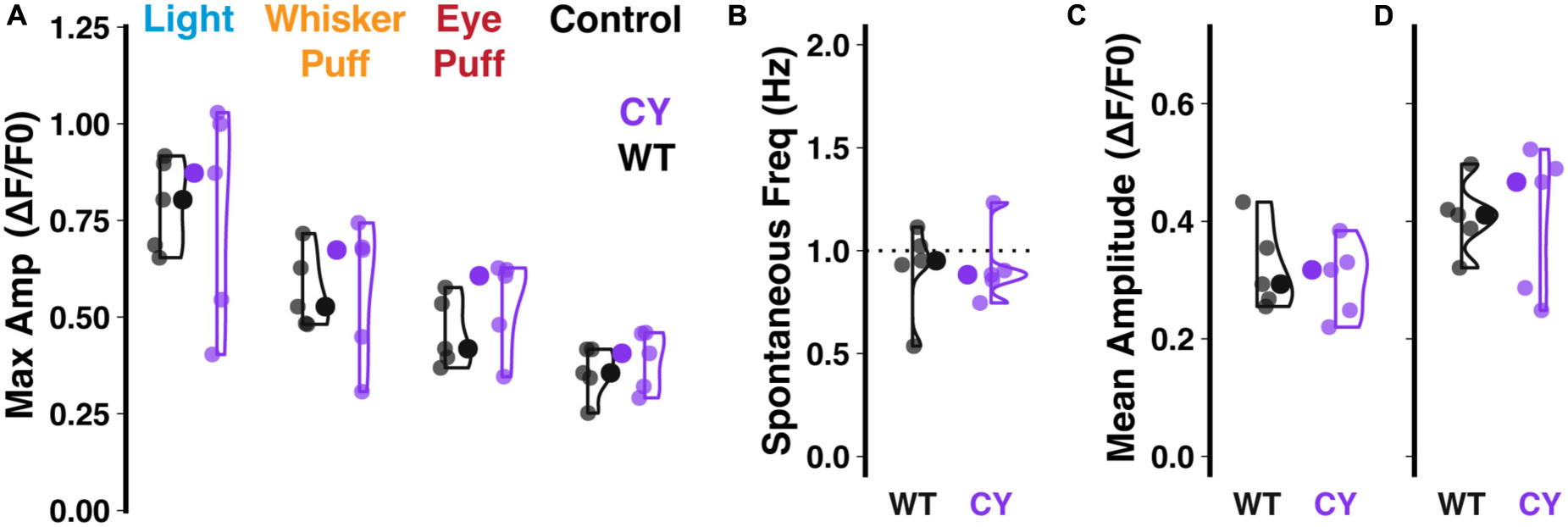

Supplement: Supplementary Figure 2 — Multisensory responsiveness averaged by animal. (A–D) All cell data from Figures 4E, H–J averaged by animal. Large inset, hollow points indicate median while smaller points depict individual values. [file Image_2.tif]

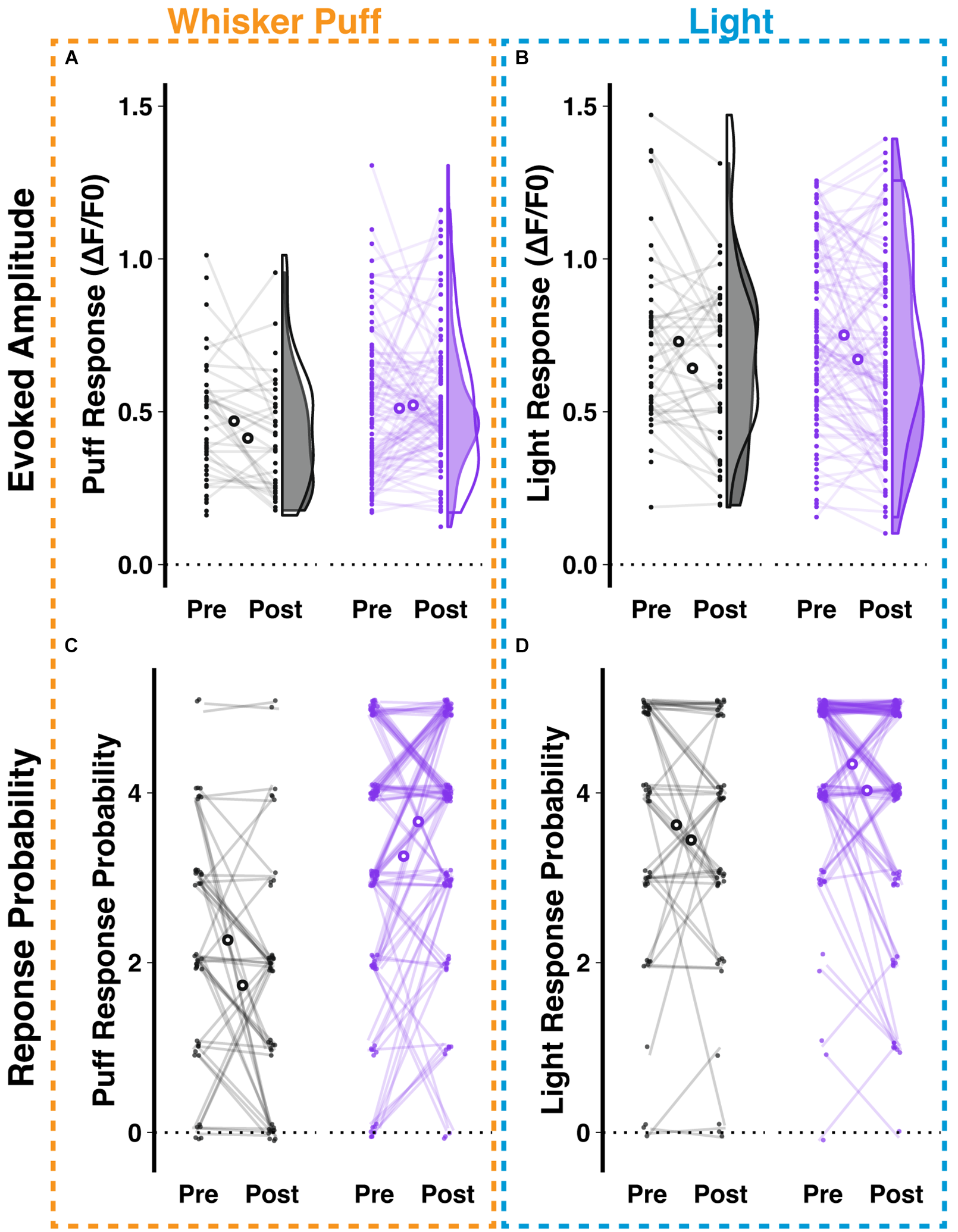

Supplement: Supplementary Figure 3 — Un-normalized evoked Ca2+ amplitude and probability of response reflects the same effect of repeated exposure as observed with normalized data despite variable baseline responsiveness. (A,B) Raw Puff and Light evoked response ΔF/F0 values for each cell pre vs. post. (C,D) Raw Puff and Light evoked response probability for each cell out of 5 trials during the first 20 min before and after repeated exposure. For (A–D), large inset, hollow points indicate mean ± SEM while smaller points depict individual values. [file Image_3.tif]

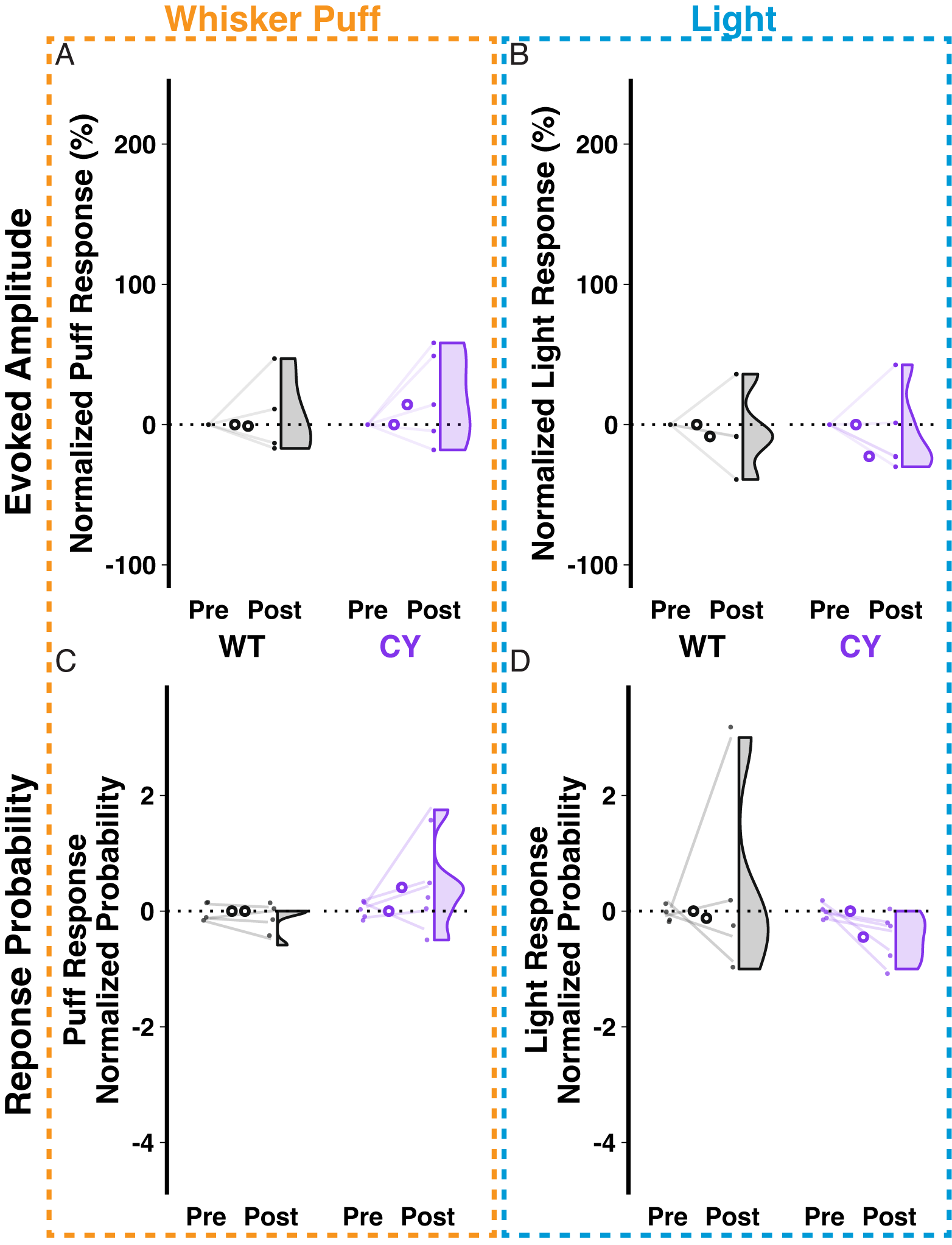

Supplement: Supplementary Figure 4 — Effects of repeated sensory exposure by animal. (A–D) As in Figures 5A–D, but with cellular data averaged across each animal. Large inset, hollow points indicate median while smaller points depict individual values. [file Image_4.TIF]

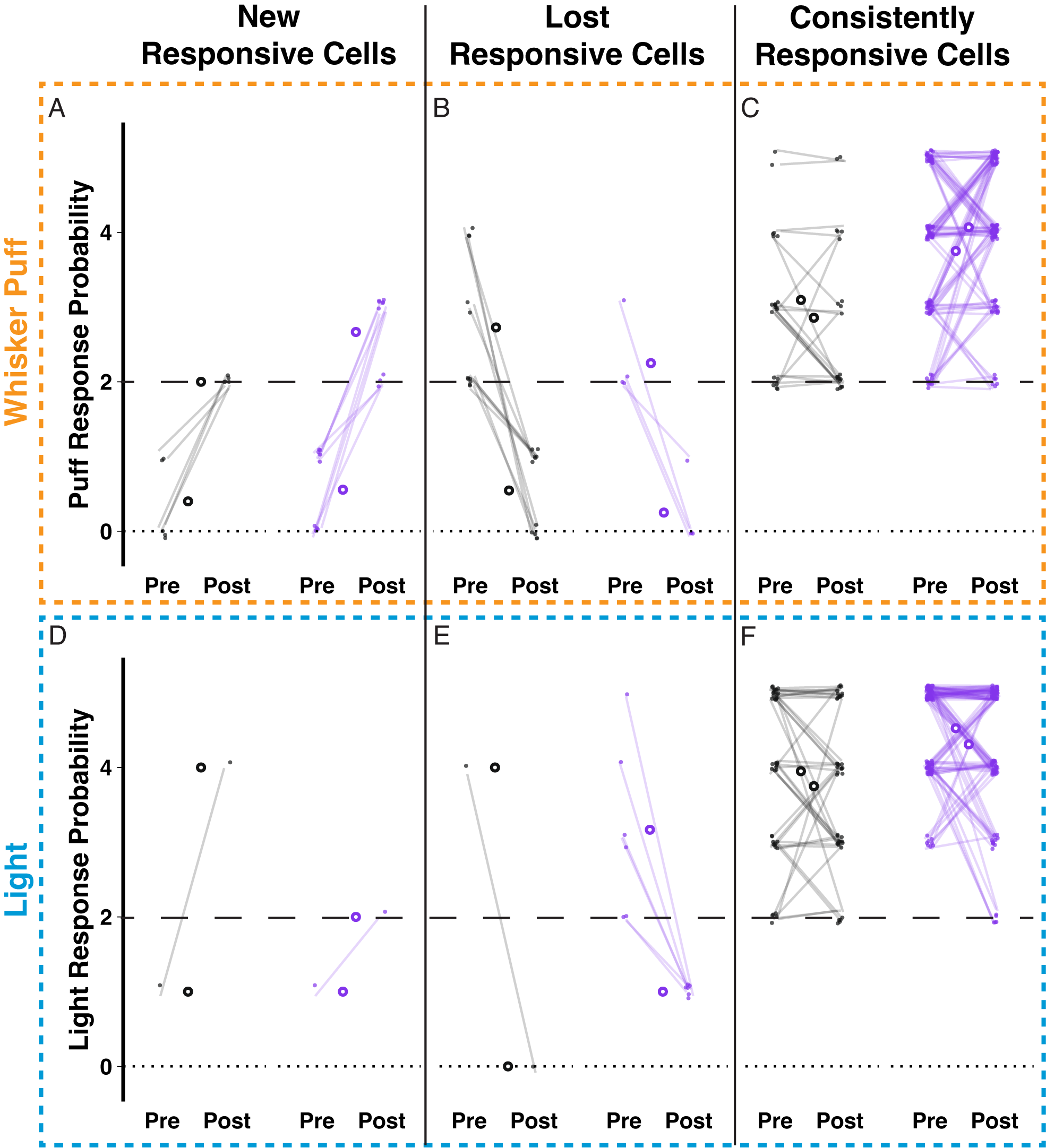

Supplement: Supplementary Figure 5 — Genotype differences in population responsiveness are supported by changes across distinct types of cellular responsiveness. (A) More unresponsive CYFIPOE cells exhibit at least moderate responsiveness following repeated exposure than WT. Here, cells are defined as responsive if they exhibit more than one Ca2+ peak within the response window across five trials. This exceeds the ∼1/5 chance that ∼1 Hz spontaneous events may be present during a 250 ms response window. (B) Fewer responsive CYFIPOE cells exhibit a loss of detectable response than among WT cells. (C) Among cells that are consistently responsive, CYFIPOE cells show an increase in probability of response as compared with a decrease among WT cells. (D–F) As in panels (A–C) for Light stimuli where there is only a moderate effect of decreased responsiveness in the CYFIPOE cell population. For (A–D), large inset, hollow points indicate mean ± SEM while smaller points depict individual values. [file Image_5.TIF]
